# CAPE suppresses migration and invasion of prostate cancer cells via activation of non-canonical Wnt signaling

**DOI:** 10.18632/oncotarget.9380

**Published:** 2016-05-15

**Authors:** Jen-Chih Tseng, Ching-Yu Lin, Liang-Chen Su, Hsiao-Hui Fu, Shiaw-Der Yang, Chih-Pin Chuu

**Affiliations:** ^1^ Institute of Molecular and Cellular Biology, National Tsing Hua University, Hsinchu, Taiwan; ^2^ Institute of Cellular and System Medicine, National Health Research Institutes, Miaoli County, Taiwan; ^3^ Graduate Program for Aging, China Medical University, Taichung City, Taiwan

**Keywords:** prostate cancer, metastasis, Wnt signaling, caffeic acid phenethyl ester, Micro-Western Array

## Abstract

Prostate cancer (PCa) was the fifth most common cancer overall in the world. More than 80% of patients died from PCa developed bone metastases. Caffeic acid phenethyl ester (CAPE) is a main bioactive component of honeybee hive propolis. Transwell and wound healing assays demonstrated that CAPE treatment suppressed the migration and invasion of PC-3 and DU-145 PCa cells. Gelatin zymography and Western blotting indicated that CAPE treatment reduced the abundance and activity of MMP-9 and MMP-2. Analysis using Micro-Western Array (MWA), a high-throughput antibody-based proteomics platform with 264 antibodies detecting signaling proteins involved in important pathways indicated that CAPE treatment induced receptor tyrosine kinase-like orphan receptor 2 (ROR2) in non-canonical Wnt signaling pathway but suppressed abundance of β-catenin, NF-κB activity, PI3K-Akt signaling, and epithelial-mesenchymal transition (EMT). Overexpression or knockdown of ROR2 suppressed or enhanced cell migration of PC-3 cells, respectively. TCF-LEF promoter binding assay revealed that CAPE treatment reduced canonical Wnt signaling. Intraperitoneal injection of CAPE reduced the metastasis of PC-3 xenografts in tail vein injection nude mice model. Immunohistochemical staining demonstrated that CAPE treatment increased abundance of ROR2 and Wnt5a but decreased protein expression of Ki67, Frizzle 4, NF-κB p65, MMP-9, Snail, β-catenin, and phosphorylation of IκBα. Clinical evidences suggested that genes affected by CAPE treatment (*CTNNB1, RELA, FZD5, DVL3, MAPK9, SNAl1, ROR2, SMAD4, NFKBIA, DUSP6*, and *PLCB3*) correlate with the aggressiveness of PCa. Our study suggested that CAPE may be a potential therapeutic agent for patients with advanced PCa.

## INTRODUCTION

Prostate cancer (PCa) ranks the 5th most common cancer in the world. Surgery is effective for localized PCa. Approximately 15–35% of PCa patient eventually develop metastasis. Bone is the most frequent site of distant prostate cancer metastases, approximately 90% of patients with metastatic PCa have skeletal lesions. Bone metastases depend on dynamic crosstalk between PCa cells, bone marrow microenvironment, osteoblasts, and osteoclasts [1]. PCa cells from primary tissue undergo an epithelial-mesenchymal transition to disseminate and acquire a bone-like phenotype to metastasize in bone tissue. Hormone ablation therapy is the standard treatment for metastatic PCa. The majority of PCa patients receiving androgen ablation therapy will ultimately develop castration-resistant prostate cancer (CRPC) within 1–3 years with a median survival time of 1–2 years. Median time to prostate-specific antigen progression was significantly shorter when bone metastases were present [2]. PCa bone metastases form osteoblastic lesions, which is characterized by increased bone production [3]. Several proteins involved in Wnt signaling are involved in prostate tumor-induced osteoblastic activity [4], indicating that small molecular targeting Wnt signaling may be effective intervention for PCa metastasis.

Caffeic acid phenethyl ester (CAPE) is a main bioactive component extracted from honeybee hive propolis. CAPE treatment ranging from 50 μM to 80 μM specifically suppresses NF-κB activity by preventing the translocation of p65 unit of NF-κB and the binding between NF-κB and DNA [5]. We previously reported that CAPE dose-dependently suppressed the proliferation of both androgen-dependent and CRPC PCa cells via induction of cell cycle arrest, inhibition of c-Myc, Akt, and Skp2, as well as activation of p21^Cip1^, p27^Kip1^, and p53 [6–8]. However, it is not clear whether CAPE can suppress the metastasis of PCa cells or not. Micro-Western Array (MWA) [9] is a high-throughput antibody-based proteomics platform composes of a GeSim Nanoplotter arrayer, a GE multiphor, and a Licor Odyssey infra-red scanner. MWA allows detecting protein expression level or phosphorylation status change of 96–384 different antibodies in 6–15 samples simultaneously. We therefore performed MWA with 264 antibodies targeting cell cycle regulation, PI3K-Akt, TGF-β, NF-κB and Wnt signaling to investigate the signaling pathways being affected by CAPE treatment. PC-3 and DU-145 cells are androgen receptor (AR)-negative prostate cancer cells derived from bone and brain metastasis of PCa cells, respectively. PC-3 and DU-145 are the most commonly used PCa cell lines for metastasis study. We therefore recruit these two cell lines to determine if CAPE may be a potential therapeutic agent for treatment of PCa metastasis. We discovered that CAPE treatment suppressed migration and invasion of PC-3 and DU-145 PCa cells via induction of non-canonical Wnt signaling and inhibition of proteins involved in epithelial-mesenchymal transition (EMT). Our finding implied that CAPE treatment is a potential intervention for PCa metastasis.

## RESULTS

### CAPE treatment suppressed migration and invasion of PCa cells *in vitro*

**Figure 1:**
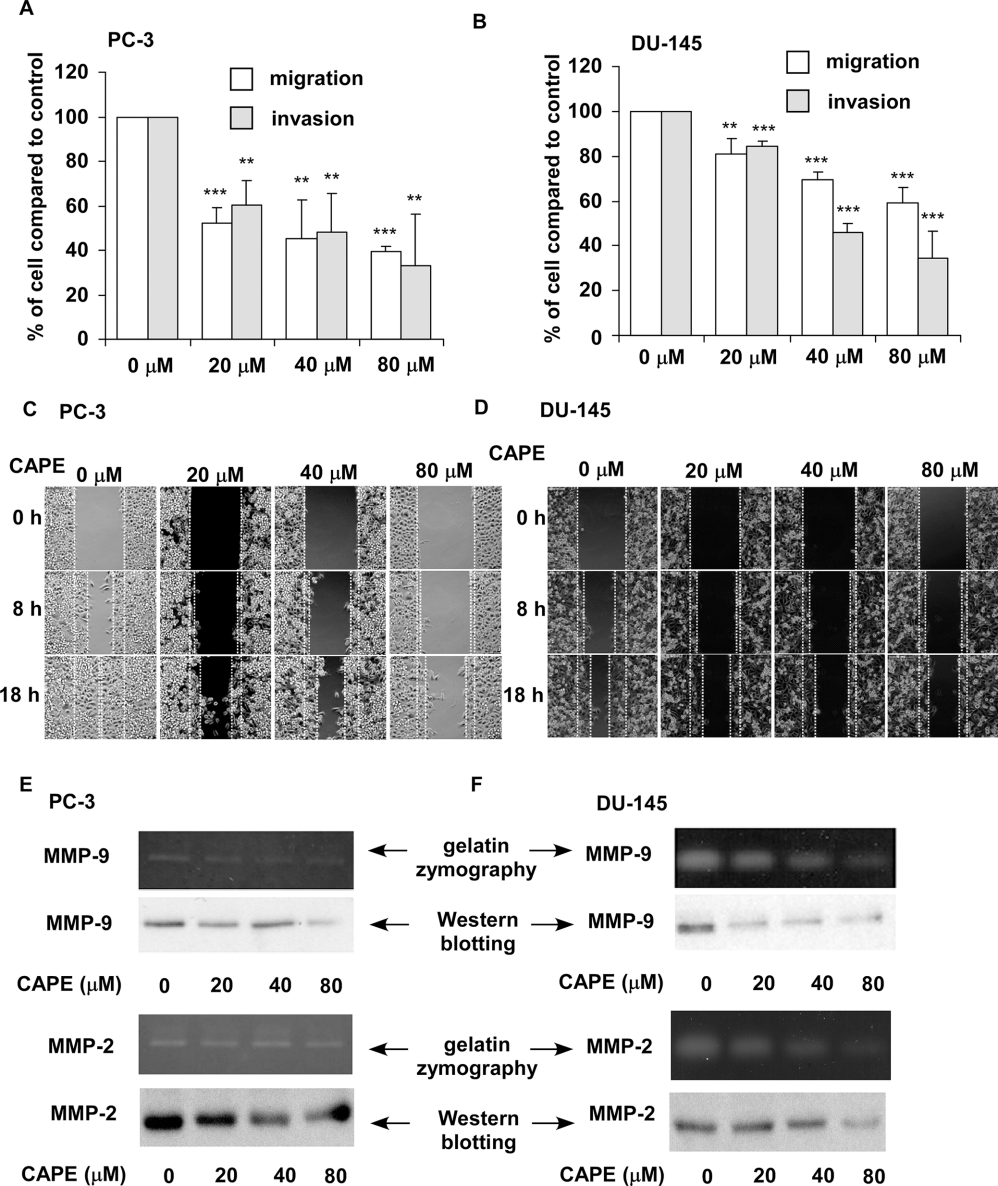
CAPE treatment suppresses migration and invasion of PC-3 and DU-145 cells *in vitro* Migration and invasion ability of PC-3 (**A**) and DU-145 (**B**) cells pre-treated with different concentration of CAPE (0, 20, 40, 80 μM) for 24 h was determined by transwell assay. Asterisk ** and *** represents statistically significant difference *p* < 0.01 and *p* < 0.001, respectively, between control and treatment groups. Motility of PC-3 (**C**) and DU-145 (**D**) cells pre-treated with different concentration of CAPE (0, 20, 40, 80 μM) for 24 h was determined by wound healing assay. Images are obtained by live imaging microscope (Leica AF 6000 LX, Leica, Wetzlar, Germany). The activity and abundance of secreted MMP-9 and MMP-2 in culture medium of PC-3 (**E**) and DU-145 (**F**) cells pre-treated with different concentration of CAPE (0, 20, 40, and 80 μM) for 24 h was determined by gelatin zymography and Western blot analysis, respectively.

CAPE pre-treatment (20, 40 and 80 μM) for 24 h dose-dependently suppressed the migration and invasion of PC-3 and DU-145 PCa cells as determined by transwell assay (Figure 1A, 1B) and wound healing assay (Figure 1C, 1D). Gelatin zymography assay (Figure 1E, 1F) and Western blotting assay (Figure 1E, 1F) revealed that CAPE treatment reduced the activity and abundance of MMP-9 and MMP-2 secreted from PC-3 and DU-145 cells.

### CAPE treatment altered the abundance and phosphorylation status of proteins involved in cell cycle regulation, PI3K-Akt signaling, NF-κB and Wnt signaling

**Figure 2:**
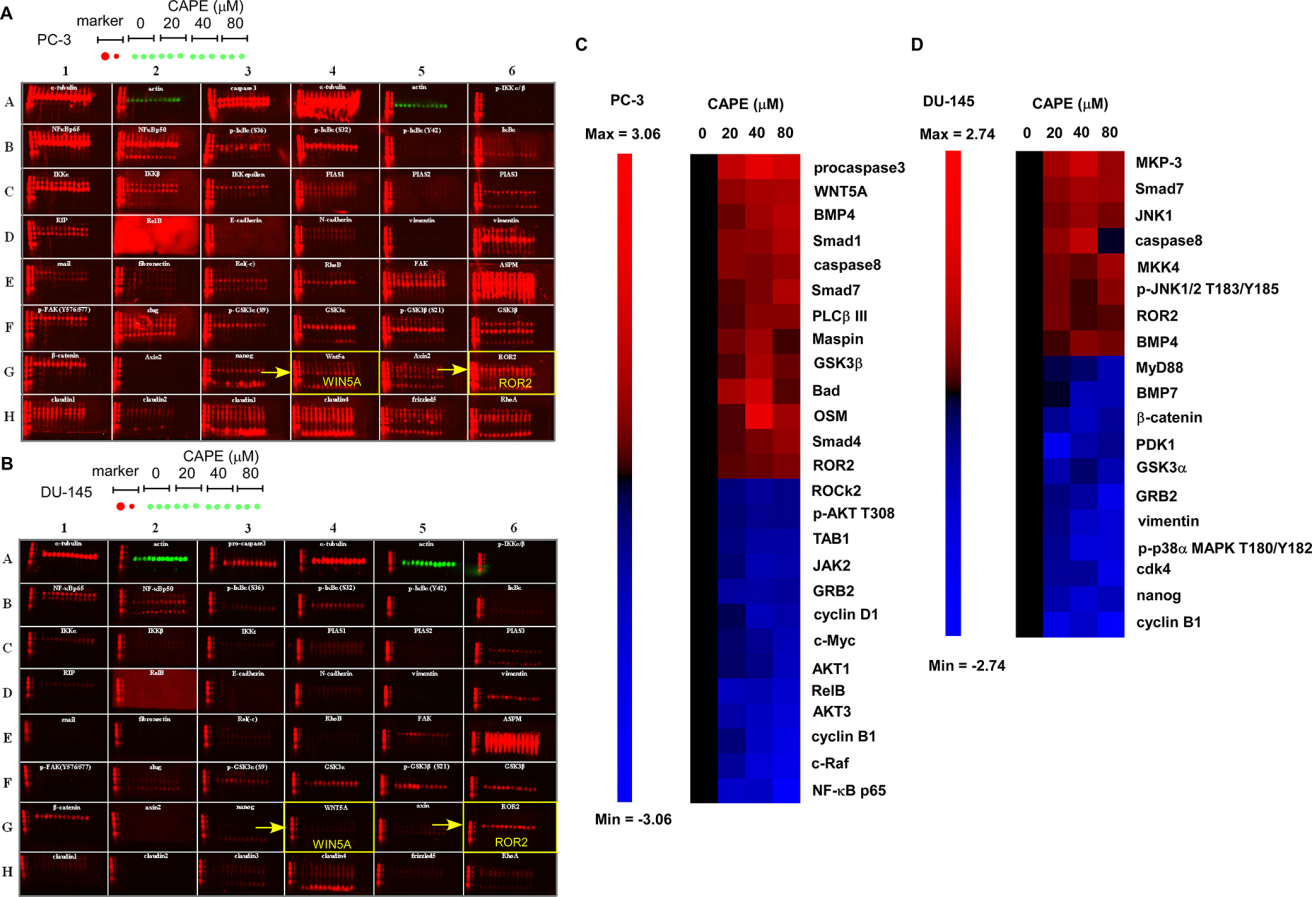
CAPE treatment affected abundance and phosphorylation status of proteins regulating cell proliferation and tumor metastasis Expression level and phosphorylation status of signaling proteins involved in cell cycle regulation, Wnt signaling, PI3K-Akt signaling, and NF-κB in PC-3 and DU-145 PCa cells treated with 0, 20, 40, 80 μM CAPE for 24 h was determined by Micro-Western Array with 264 different antibodies. A representative image of PC-3 and DU-145 PCa cells being assayed with Micro-Western Array was shown, respectively. Proteins with expression level increased or decreased at least 1.5 fold under CAPE treatment in PC-3 (**C**) and DU-145 (**D**) cells were demonstrated by heatmap.

We performed Micro-Western Array (MWA) to investigate the signaling proteins being affected by CAPE treatment. Protein expression profile in PC-3 and DU-145 cells being pre-treated with 0, 20, 40, or 80 μM CAPE for 24 h was determined by MWA with 264 different antibodies detecting important signaling proteins (Figure 2A, 2B). In PC-3 cells, CAPE significantly increased protein abundance of pro-caspase 3, Wnt5a, BMP4, Smad1, Caspase 8, PLCβ III, Maspin, GSK-3β, Bad, Smad4, ROR2, but decreased protein levels of NF-κB p65, c-Raf, Cyclin B1, Akt3, RelB, Akt1, c-Myc, Cyclin D1, Jak2, TAB2, phospho-Akt Thr308, and ROCK2 (Figure 2C). In DU-145 cells, CAPE treatment significantly elevated the protein abundance of MKP-3, Smad7, JNK1, Caspase 8, MKK4, phospho-JNK1/2 Tyr 185/Thr183, ROR2, and BMP4 but lessened the protein levels of Cyclin B1, nanog, Cdk4, phosphor-p38a MAPK Thr180/Tyr182, vimentin, GRB2, GSK-3α, PDK1, β-catenin, BMP7, and MyD88 (Figure 2D).

**Figure 3:**
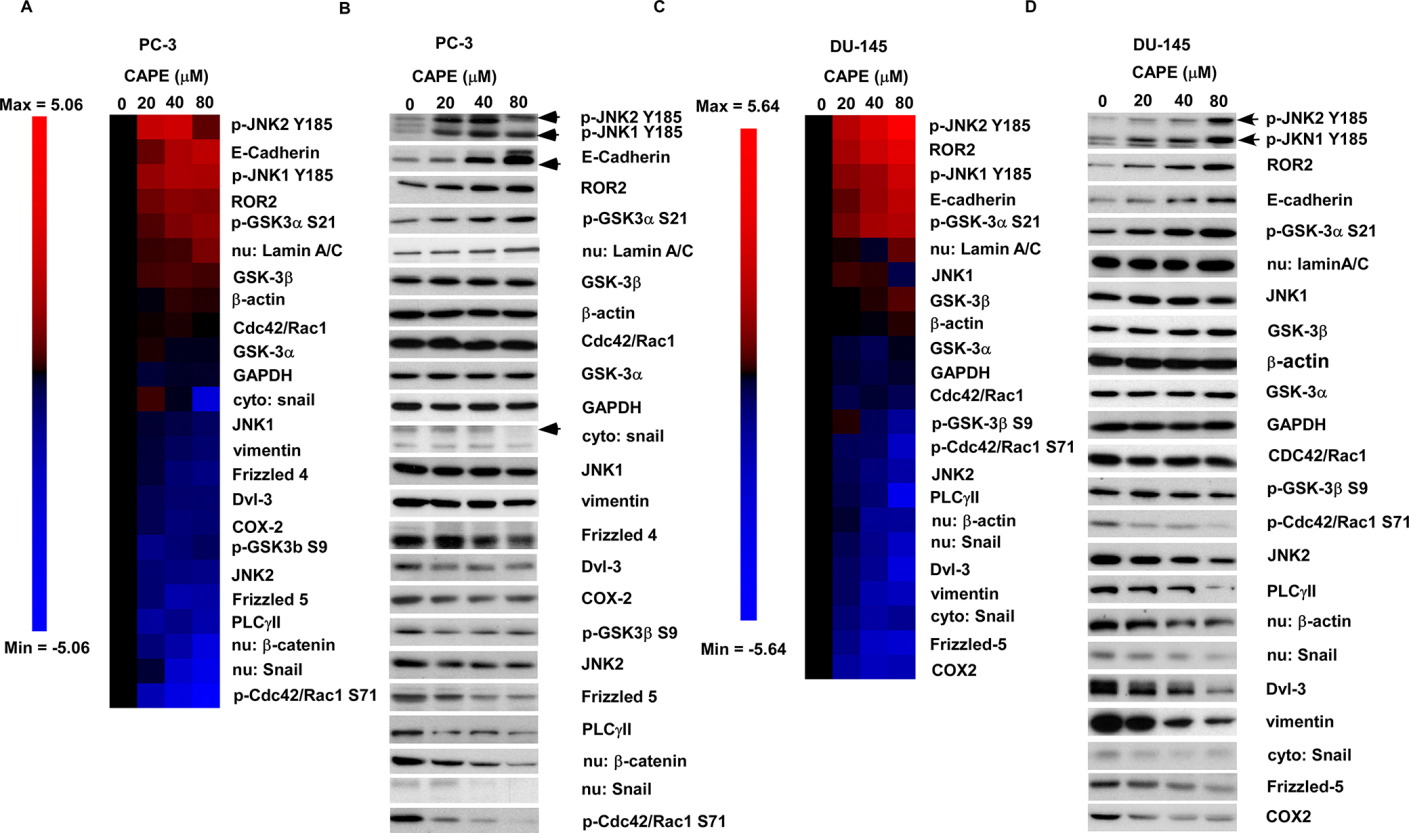
CAPE treatment suppressed expression of signaling proteins involved in canonical Wnt signaling and EMT but induced expression of signaling proteins in non-canonical Wnt signaling Heatmap illustration and Western blotting images of the expression levels and phosphorylation status of signaling proteins regulating canonical Wnt Signaling, non-canonical Wnt signaling and EMT in PC-3 cells (**A**–**B**) and DU-145 (**C**–**D**) treated with different concentrations of CAPE for 24 h were determined by conventional Western blotting assay. Expression of β-actin was used as loading control for whole cell lysate. Expression of Lamin A/C and GAPDH was used as loading control for nuclear and cytoplasmic protein extract, respectively, in nuclear-cytoplasmic separation lysate.

As MWA analysis indicated that several signaling proteins involved in Wnt signaling were affected by CAPE treatment and Wnt signaling plays essential role in regulating prostate cancer metastasis, we used conventional Western blotting to evaluate the effects of CAPE on proteins regulating Wnt signaling and epithelial mesenchymal transition (EMT). EMT plays important role in cancer invasion and metastases [10, 11]. In PC-3 cells, CAPE treatment increased protein expression level of phospho-JNK2 Tyr185, E-Cadherin, phospho-JNK1 Tyr185, ROR2, phospho-GSK-3a Ser21, GSK-3β, but decreased abundance of phospho-Cdc42/Rac1 Ser 71, nuclear Snail, nuclear β-catenin, PLC-g II, Frizzled 5, JNK2, phospho-GSK-3β Ser9, Cox-2, Dvl-3, Frizzled 4, vimentin, cytoplasmic Snail, and JNK1 (Figure 3A, 3B). In DU-145 cells, CAPE treatment elevated protein abundance of phospho-JNK2 Tyr185, ROR2, phospho-JNK1 Tyr185, E-Cadherin, phospho-GSK-3a Ser21, and GSK-3β, but reduced protein levels of Cox-2, Frizzled 5, cytoplasmic Snail, vimentin, Dvl-3, nuclear Snail, nuclear β-catenin, PLC-g II, JNK2, phospho-Cdc42/Rac1 Ser 71, phospho-GSK-3β Ser9, and Cdc42/Rac1 (Figure 3C, 3D).

### CAPE treatment suppressed EMT through elevation of ROR2 and reduction of β-catenin-dependent signaling

**Figure 4:**
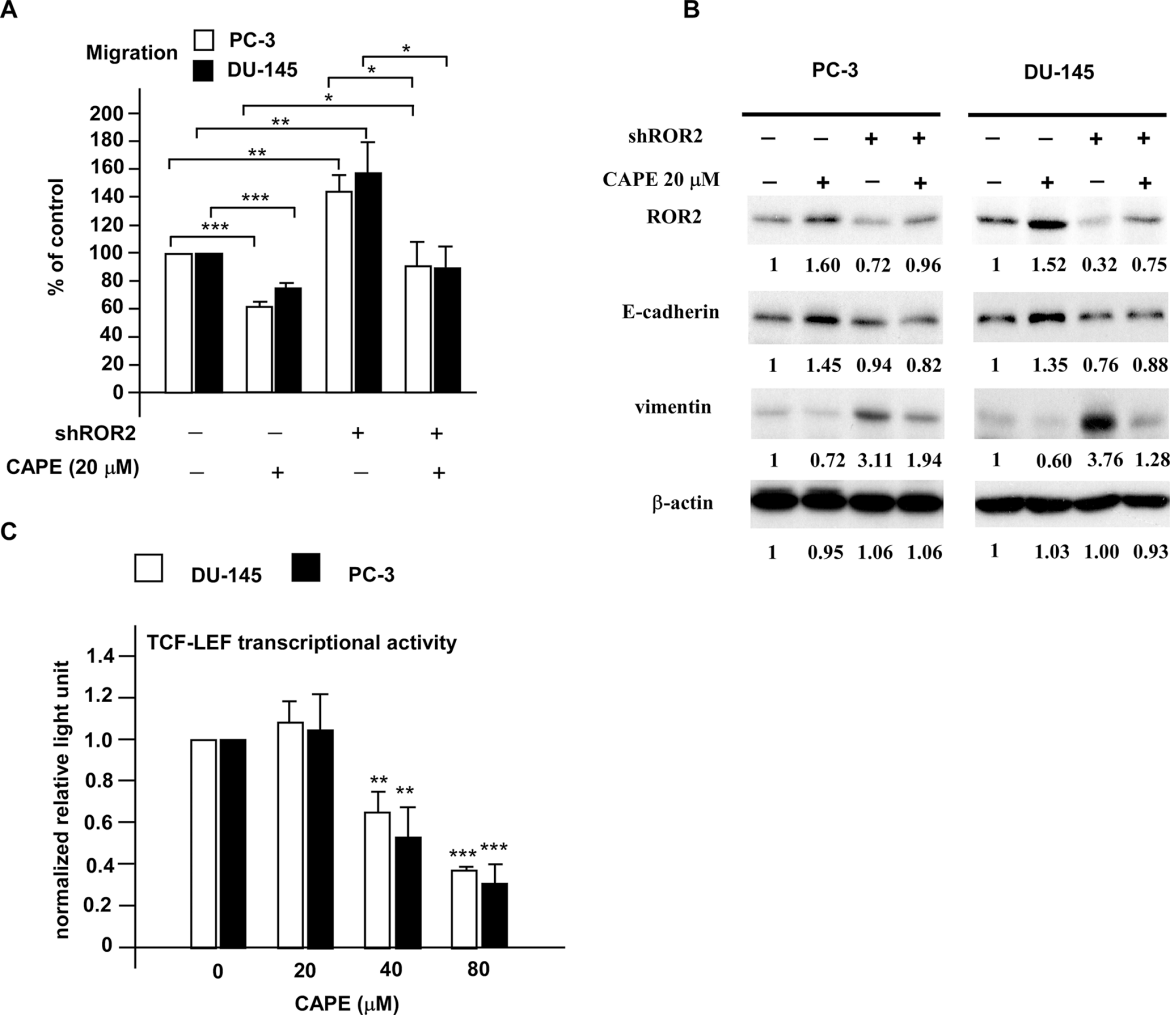
CAPE treatment suppressed migration of PC-3 and DU-145 PCa cells via induction of ROR2 as well as suppression of EMT and canonical Wnt signaling (**A**) Migration of DU-145 and PC-3 PCa cells transfected with shROR2 or control vector were treated with or without 20 μM CAPE for 24 h. Migration ability of cells were determined by transwell assay. (**B**) Proteins levels of ROR2, E-cadherin and vimentin in DU-145 and PC-3 PCa cells with or without shROR2 knockdown in the presence or absence of 20 μM were determined by Western blotting assay. Expression of β-actin was used as loading control. (**C**) DU-145 and PC-3 cells were co-transfected with vector containing eight copies of a TCF-LEF response element driving the transcription of the luciferase reporter gene luc2P and phRL-CMV-Renilla luciferase vector. Cells were treated with 0, 20, 40, or 80 μM CAPE for 24 h. Intensity of luciferase was measured with Dual-Luciferase kit (Promega) and a Monolight luminometer to determine the canonical Wnt signaling. Asterisks *, **, and *** represent statistically significant difference *p* < 0.05, *p* < 0.01, and *p* < 0.001, respectively, between the groups being compared.

ROR2 receptor and Wnt5a are involved in non-canonical Wnt signaling pathway. As CAPE treatment stimulated protein abundance of ROR2 and Wnt5a in PCa cells, we determined if alteration of ROR2 level may affect migration of PC-3 and DU-145 cells. As shown in Figure 4A, knockdown of ROR2 by shRNA significantly enhanced the migration of PC-3 and DU-145 cells, while treatment with 20 μM CAPE significantly suppressed the migration of PC-3 and DU-145 cells. Knockdown of ROR2 by shRNA blocked the suppressive effects of CAPE on prostate cancer cell migration. These results indicated that CAPE treatment suppressed prostate cancer cell migration via induction of ROR2. Elevation of ROR2 and E-Cadherin proteins and reduction of vimentin protein caused by CAPE treatment in PC-3 and DU-145 cells were reversed by shRNA knockdown of ROR2 (Figure 4B).

Activation of Wnt signaling pathway causes the dephosphorylation, stabilization, and nuclear translocation of β-catenin. The stabilized β-catenin will then complex with TCF/LEF, a transcription factor, resulting in the activation of canonical Wnt signaling downstream genes. CAPE treatment dose-dependently suppressed the TCF-LEF transcriptional activity in DU-145 and PC-3 cells (Figure 4C). These results indicated that CAPE treatment suppressed prostate cancer cell migration, at least partially, via inhibition of canonical Wnt signaling.

### CAPE treatment suppressed metastasis of PC-3 PCa cells in nude mice

**Figure 5:**
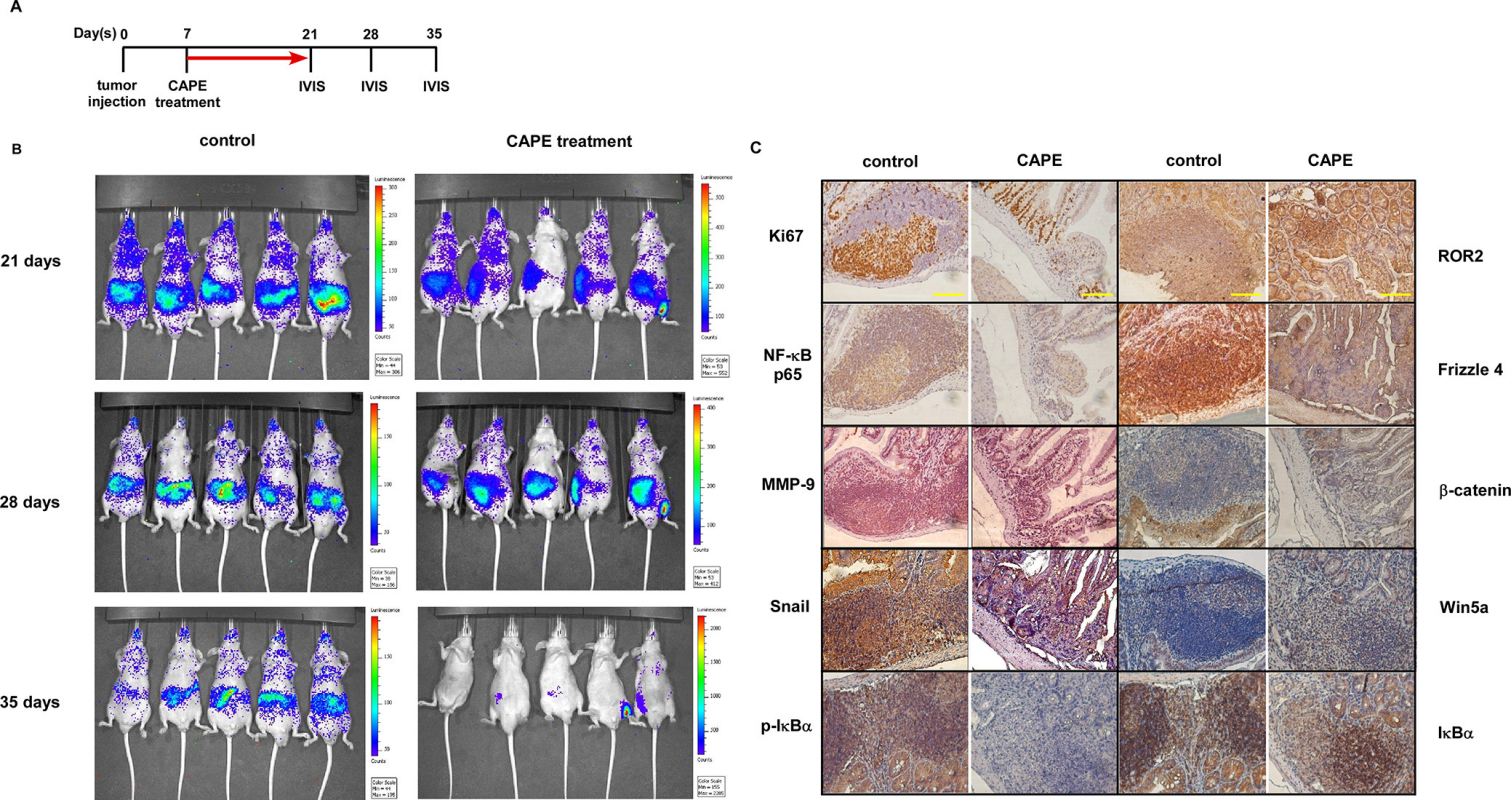
CAPE treatment suppressed cancer metastasis of PC-3 xenografts in nude mice (**A**) Experimental schedule of PC-3^luc^ xenografts tail vein injection model treated with CAPE. (**B**) PC-3^luc^ cells (1 × 10^6^) were injected into tail vein of 6-8 weeks nude mice. Mice carrying PC-3^luc^ tumors were separated into CAPE treatment group (5 mice) and vehicle control group (5 mice). Mice were treated with control vehicle or CAPE (15 mg/kg) via i.p. injection twice a week for one month. The distribution and migration of PC-3^luc^ cells was monitored weekly by *in vivo* bioluminescence IVIS imaging system. Images of 3rd, 4th, and 5th week were shown. (**C**) Immunohistochemistry staining was performed to determine levels of Ki67, NF-κB p65, MMP-9, Snail, phospho-IκBα Ser32, ROR2, Frizzle 4, β-catenin, Wnt5a, and IκBα proteins in PC-3 tumors. Scale bars represent 100 μm at 400× magnification.

In order to evaluate the possibility of using CAPE to suppress prostate cancer metastasis, we injected PC-3^luc^ carrying firefly luciferase-reporter gene into nude mice tail vein through intravenous injection. The expansion and migration of PC-3^luc^ PCa cells was monitored by living image IVIS system (Figure 5A). Treatment with CAPE (15 mg/kg, twice a week) for one month significantly reduced the metastasis of PC-3^luc^ xenografts as compared to the control group (Figure 5B).

We observed that PC-3^luc^ PCa cells in control group migrated to small intestine and were positively stained with Ki67 while PC-3^luc^ PCa cells in CAPE treatment group migrated poorly and contained very little cells positively stained with Ki67 (Figure 5C). CAPE treatment also suppressed the expansion of PC-3^luc^ cells to lung and lymph node (Supplementary Figure 1A and 1B). IHC staining indicated that CAPE treatment decreased protein expression of NF-κB p65, MMP-9, Snail, β-catenin, Frizzled 4, and phosphorylation of IκBα (Figure 5C) but increased the protein abundance of ROR2 and Wnt5a (Figure 5C).

### Clinical significance of genes associated with metastasis was affected by CAPE treatment

**Figure 6:**
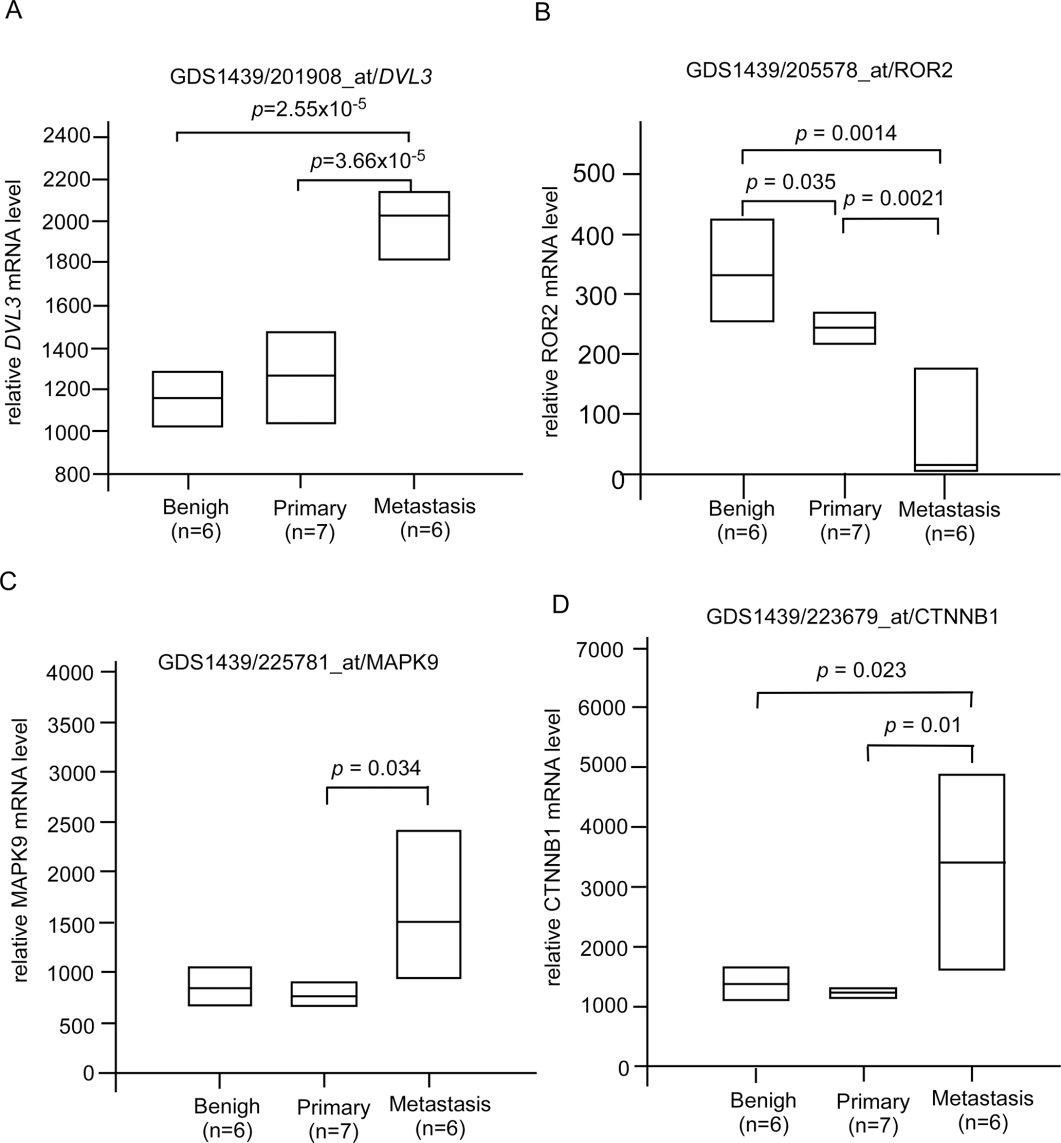
Clinical significance of genes affected by CAPE treatment in GEO Profile GDS1439 dataset The mRNA level of (**A**) *DVL3*, (**B**) *ROR2*, (**C**) *MAPK9*, (**D**) CTNNB1 in primary vs. metastatic prostate tumors was analyzed from online gene array data PubMed GEO Profile GDS1439 dataset.

**Figure 7:**
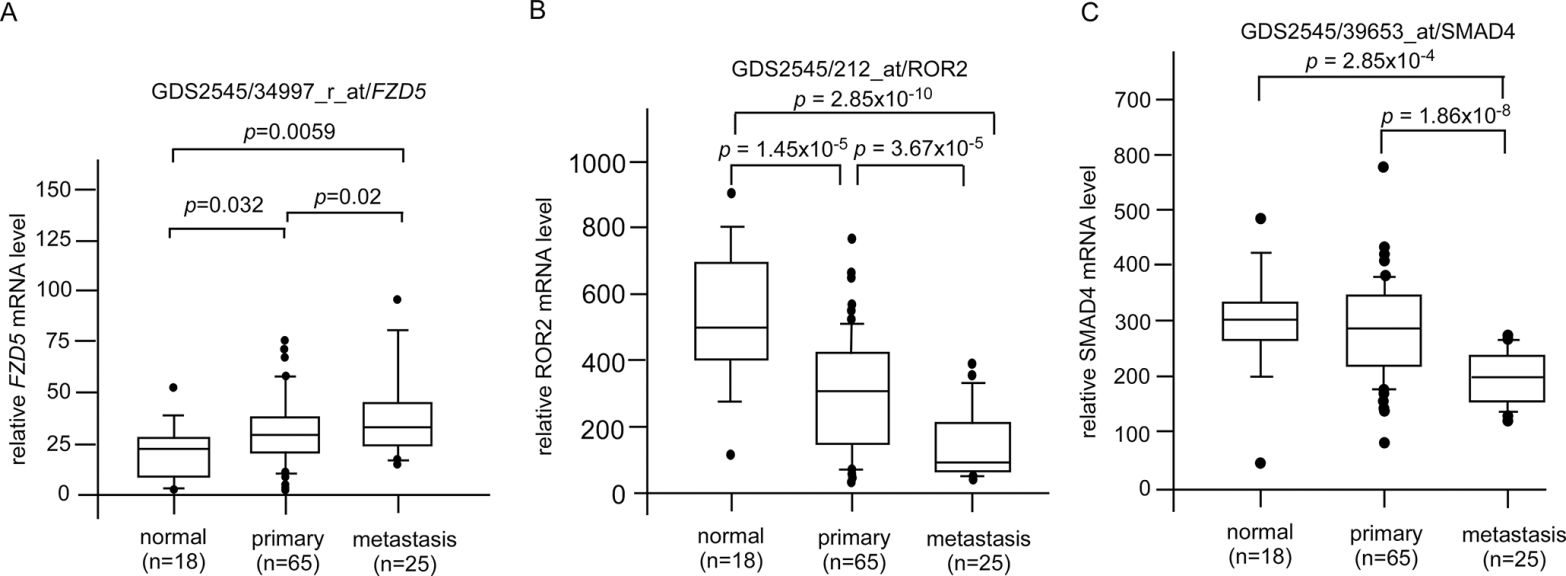
Clinical significance of genes affected by CAPE treatment in GEO Profile GDS2545 dataset The mRNA level of (**A**) *FZD5*, (**B**) *ROR2*, (**C**) *SMAD4* in primary vs. metastatic prostate tumors was analyzed from online gene array data PubMed GEO Profile GDS2545 dataset.

**Figure 8:**
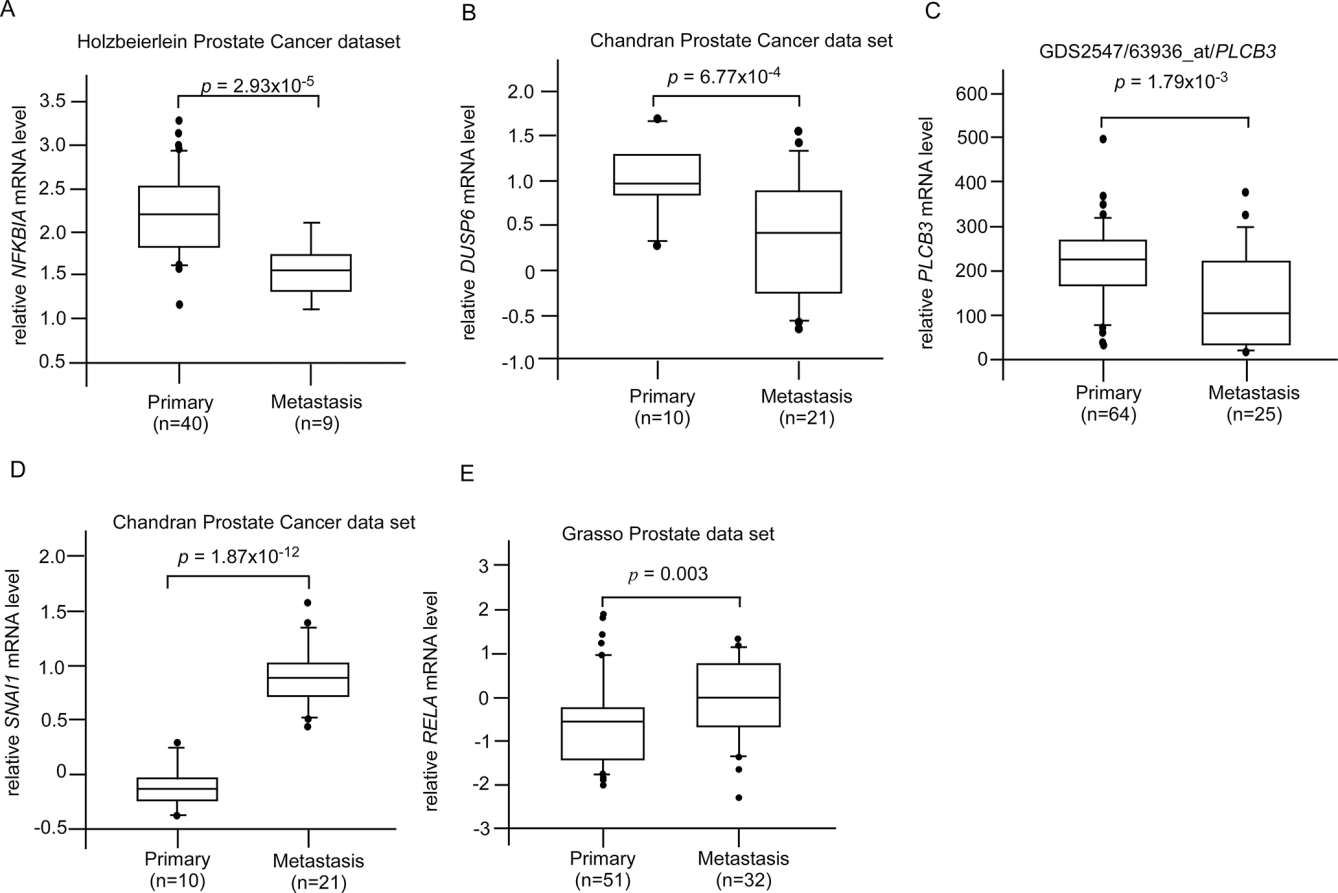
Clinical significance of genes affected by CAPE treatment in Oncomine dataset The mRNA level of (**A**) *NKKBIA*, (**B**) *DUSP6*, (**C**) *PLCB3*, (**D**) *SMAD4*, (**E**) *RELA*, and (**F**) *SNAI1* in primary vs. metastatic prostate tumors was analyzed from online gene array data extracted from both Oncomine and PubMed GEO Profile.

CAPE treatment suppressed JNK2, β-catenin, and DVL3 but increased ROR2 protein expression (Figures 2, 3, 5). These proteins are involved in Wnt signaling. We determined the clinical significance of DVL3 (Figure 6A), ROR2 (Figure 6B), MAPK9 (JNK2) (Figure 6C), and CTNNB1 (β-catenin) (Figure 6D) by analyzing online database in PubMed GEO profile GDS1439 dataset. Compared to the normal prostate tissues or primary prostate tumors, the mRNA levels of *DVL3*, MAPK9, and CTNNB1 are higher in metastatic prostate tumors while the mRNA level of ROR2 is lower in metastatic prostate tumors (Figure 6). CAPE treatment increased Smad4 (Figure 2) but decreased Frizzled 5 (Figure 3). PubMed GEO profile GDS2545 dataset indicates that FZD5 (Frizzled 5, Figure 7A) is higher in metastatic prostate tumors while the mRNA level of ROR2 (Figure 7B) and *SMAD4* (Figure 7C) are lower in metastatic prostate tumors. Additionally, CAPE treatment reduced abundance of NF-κB, Snail, Frizzled 4 as well as phosphorylation of IκBα (Figure 3, Figure 5), which are encoded by *RELA*, *SNAI1*, *FZD4*, and *NFKBIA* gene, respectively. On the other hand, CAPE treatment increased MKP-3 (Mitogen-Activated Protein Kinase Phosphatase 3) and PLC-β III, which are encoded by *DUSP6* and *PLCB3* gene, respectively (Figure 2). Compared to the primary prostate tumors, mRNA level of *NFKBIA* (Figure 8A), *DUSP6* (Figure 8B), *PLCB3* (Figure 8C), and *SMAD4* (Figure 8D) are lower in metastatic prostate tumors, while mRNA level of *RELA* (Figure 8E) and *SNAI1* (Figure 8F) exhibit opposite trend.

## DISCUSSION

In the canonical Wnt pathway, Wnt ligands bind to Frizzled receptors and low-density lipoprotein receptor-related protein 5/6 (LRP5/6), leading to activation of disheveled (Dsh), inhibition of glycogen synthase kinase-3β (GSK-3β), and disaggregation of adenomatous polyposis coli (APC), Axin, and GSK-3β [12, 13]. Activation of canonical Wnt signaling causes the stabilization, cytoplasmic accumulation, and nuclear translocation of β-catenin [12, 13]. The β-catenin binds to lymphoid enhancer factor (LEF)/T-cell factor (TCF) in nucleus and induces transcription of Wnt target genes [12, 13]. On the other hand, Wnt5a is identified as a non-canonical Wnt family member. Wnt5a inhibits Wnt3a protein-induced canonical Wnt signaling [14]. The Wnt5a signal is mediated either by the orphan tyrosine kinase ROR2 or Frizzled receptor [15]. Wnt5a inhibits β-catenin signaling when binds to the ROR2, while Wnt5a activates β-catenin signaling when binds with Frizzled receptors [16].

Wnt signaling plays essential role in regulation of PCa metastasis. PCa bone metastases form osteoblastic lesions, which is characterized by the increase of bone production [3]. Families of soluble frizzled related receptors [sFRP), the secreted Wnt antagonists, and dickkopfs [DKK) proteins, the Wnt inhibitor, are involved in prostate tumor-induced osteoblastic activity [4]. Production of DKK-1, mediated by receptor activator of NF-κB ligand [RANKL) [17] and noggin [18], inhibitor of transforming growth factor-β [TGF-β), happens in the early stage of PCa bone metastases development. DKK-1 blocked Wnt activation of the bone morphogenetic proteins (BMP) promoter, resulting in inhibition of osteogenic Wnts and stimulation of osteolysis at the metastatic sites. During progression of PCa bone metastases, expression of DKK-1 decreases. This allows the up-regulation of osteoblastic activity induced by paracrine of Wnt signaling proteins produced by PCa cells and finally resulting in osteosclerosis at the metastatic site [17]. Knockdown of BMP expression in C4-2B cells inhibited Wnt-induced osteoblastic activity [19]. Knockdown and overexpression of Wnt5a in human prostate cancer cell lines reduced and stimulated the invasion activities of PCa cells, respectively [16, 20]. The regulation of PCa cell invasion by Wnt5a required Frizzled2 and ROR2 as Wnt receptors [16, 20].

In this study, we demonstrated that CAPE treatment dose-dependently suppressed the migration and invasion of PC-3 and DU-145 PCa cells (Figure 1). We observed that signaling proteins ROR2, Wnt5a and phospho-JNK, which belonged to the non-canonical Wnt signaling, were up-regulated in CAPE treated DU-145 and PC-3 cells 9 Figures 2,3). CAPE treatment activated ROR2 activity and thus stimulated the non-canonical Wnt signaling pathway (Figure 4). However, the β-catenin dependent signaling [nuclear level of β-catenin, c-Myc and cyclin D1) was suppressed by CAPE treatment. Additionally, CAPE treatment inhibited the Wnt3a-induced canonical pathway. The inhibition of canonical pathway was evidenced by the reduction of Frizzled receptor and its cytoplasmic mediators of Dvl-3 as well as decrease in nuclear β-catenin and TCF-LEF transcriptional activity (Figure 4). Induction of non-canonical Wnt signaling and inhibition of canonical Wnt signaling suppressed of the migration and invasion of PCa cells. CAPE treatment suppressed abundance of JNK2 but increased the phosphorylation of JNK2 (Figure 3). We believe that this is because that CAPE treatment reduces abundance and activity of NF-κB while elevates abundance of ROR2 ad Wnt5a. This in turn then increased the phosphorylation of JNK1/2, which decreased the phosphorylation of GSK-3β. Expression level of cyclin B1, cyclin D1, Cdk4, Grb2, c-Myc and MEK-5 involving in cell cycle regulation were down-regulated by CAPE treatment. The Akt signaling proteins (Akt1, Akt3, phospho-Akt Ser473 and Thr308) were significantly suppressed in CAPE treated PC-3 cells. PTEN was an inhibitor for PI3K-Akt signaling and mutation or deletion of PTEN was observed in the majority of PCa patient. PC-3 is a PTEN null PCa cell line, suggesting that CAPE might be beneficial for PCa patient with PTEN mutation or AKT activation.

Abnormal activation of canonical β-catenin dependent-WNT signaling in cancer cells resulting in induced epithelial-mesenchymal transition (EMT) and enhanced stem like properties of cancer cells [21]. Inactivation of LRP5 resulted in mesenchymal to epithelial transition (MET), decrease of translocation of β-catenin to cell surface, and inhibition of migration and invasion in PCa cells [22]. EMT, essential for cancer metastasis, is a process reducing cell polarity and cell-cell adhesion. EMT converts cells to mesenchymal phenotype, promotes migratory and invasive ability, increases apoptosis resistance, and augments production of ECM components of cells. EMT plays essential role in regulation of metastasis and diseases progression of prostate cancer [23]. During EMT, expression of several proteins change, including the up-regulation of N-Cadherin, vimentin, Snail, Slug, Twist, MMP-2, MMP-3, and MMP-9 proteins as well as down-regulation of E-Cadherin, cytokeratin, and occluding proteins. In addition, the activity of ILK, GSK-3β, and Rho as well as nuclear β-catenin, Smad-2/3, NF-κB, Snail, Slug, and Twist proteins also increased during EMT [24].

EMT regulatory proteins play essential roles in regulation of PCa metastasis. Expression of the E-Cadherin is reduced or absent in high-grade PCa [25] while reduced E-Cadherin level correlates to metastasis and lower survival in PCa patients [26, 27]. N-Cadherin elevates after hormone ablation therapy and is associated with metastasis and aggressiveness of PCa [28, 29]. MMP-9, usually up-regulated during EMT in cancer cells, is a matrixin belongs to the zinc-metalloproteinases family degrading the extracellular matrix, which is important for angiogenesis, wound healing, cell migration, and angiogenesis [30]. Elevated of MMP-9 correlates to the invasive capability of PCa cells [31, 32]. Slug is a zinc-finger transcription factor of the Snail/Slug zinc-finger family regulating cancer metastasis [33]. Slug has been reported to promote migration and invasion of PCa cells via CXCR4/CXCL12 axis [33]. Snail locates proximal to the transcriptional start site of the E-cadherin gene and it belongs to a family of ZINC-FINGER-containing transcriptional repressors [34–36]. Snail is a repressor of RKIP transcription and expression of Snail and Slug suppress the expression of E-Cadherin [33–36]. Elevation of vimentin protein, a type III intermediate filament protein, positively correlates with the invasion and metastasis potential of androgen-independent PCa cells [37]. Down-regulation of β-catenin abundance and GSK-3β phosphorylation correlates to reduced cellular migration and invasion in PCa cells [38]. GSK-3β inhibition depletes the population of PCa stem cells and attenuates the growth of metastatic prostate tumors [39]. Our observed that CAPE treatment reduced activity of MMP-9 as well as abundance of MMP-9, vimentin, Snail, and phosphorylation of GSK-3β but elevated epithelial marker of E-Cadherin and GSK-3β, suggesting that CAPE treatment suppressed PCa metastasis via inhibition of Wnt signaling and EMT (Figures 2–5).

Protein levels of β-catenin is higher in human PCa cell line as compared to normal prostate cells [40]. Cytoplasmic and nuclear β-catenin expression was detected by IHC staining in 34% of primary prostate carcinoma specimens, whereas normal prostatic tissue failed to exhibit any detectable nuclear staining for β-catenin [40]. Elevation of β-catenin protein expression was observed in 77% of lymph node metastases and in 85% of bone metastases, and high expression level of β-catenin was related directly to the Gleason score and to serum PSA levels in PCa patients [40]. Elevation of β-catenin and androgen receptor (AR) protein expression correlated with high Gleason grade, disease progression, and increasing serum prostate-specific antigen (PSA) levels in PCa patients [41]. The intensity of membrane associated β-catenin switched to cytoplasm was used to predict the higher risk of death in prostate cancer patients [42]. Our results revealed that CAPE treatment can significantly suppressed total and nuclear abundance of β-catenin (Figures 2, 3, 5), which may contribute to the inhibition of PCa metastasis caused by CAPE treatment.

NF-κB/relA transcription factor is constitutively activated in human PCa cells and inhibition of NF-κB activity in PCa cells associates with suppression of angiogenesis, invasion, and metastasis. The IκBα (inhibitor of kappa B) inactivates the transcription of NF-κB by masking the nuclear localization signals of NF-κB proteins and thus keeps them inactive in the cytoplasm. IKK phosphorylates the inhibitory IκBα protein [43]. This phosphorylation results in the dissociation of IκBα from NF-κB, and NF-κB is therefore able to move into the nucleus and initiates gene transcription. Consistent with the well known fact that CAPE is a NF-κB specific inhibitor, our study inducated that CAPE reduces expression of NF-κB p65 and RelB in PC-3. Our animal study also revealed that CAPE treatment reduced protein abundance of NF-κB p65 in PC-3 tumors.

Our nude mice study indicated that i.p. injection of CAPE (15 mg/kg, twice a week for one month) significantly decreased the metastasis of PC-3 tumors (Figure 5). CAPE treatment reduced the abundance of Ki67, MMP-9, Snail, Frizzled4, β-catenin, and ROR2 in PC-3 tumors (Figure 5). These results indicated that CAPE may have therapeutic effects for patients with advanced PCa. As mice were treated with 15mg/kg CAPE via i.p. injection twice a week for one month. This meant that each mouse received 15 (mg/kg) × 0.025 (kg/mice) × 2 (times/week) = 0.75 mg/week. Although the dose effective for human being requires clinical trial, we try to estimate the approximate amount of CAPE required to exhibit anticancer effects. For a patient of 70 kg, the patient may require 15 (mg/kg) × 70 (kg) × 2 (times/week) = 2100 mg of CAPE per week.

Genes affected by CAPE treatment obviously correlate to PCa metastasis. Gene expression of *FZD5*, *DVL3*, *RELA*, *MAPK9*, *CTNNB1*, and *SNAI1* is higher in metastatic prostate tumors as compared to primary prostate tumors (Figure 6). On the other hand, mRNA levels of *ROR2*, *NFKBIA*, *DUSP6*, *PLCB3*, and *SMAD4* are lower in metastatic prostate tumors as compared to primary prostate tumors (Figures 6–8). As CAPE treatment suppressed protein expression of Frizzled 5, Dvl-3, NF-κB, JNK2, β-catennin, and Snail but augmented protein abundance of ROR2, IκBα, MKP-3, PLCβ III, and Smad4 (Figures 2, 3), CAPE treatment may benefit patient with metastatic PCa.

**Figure 9:**
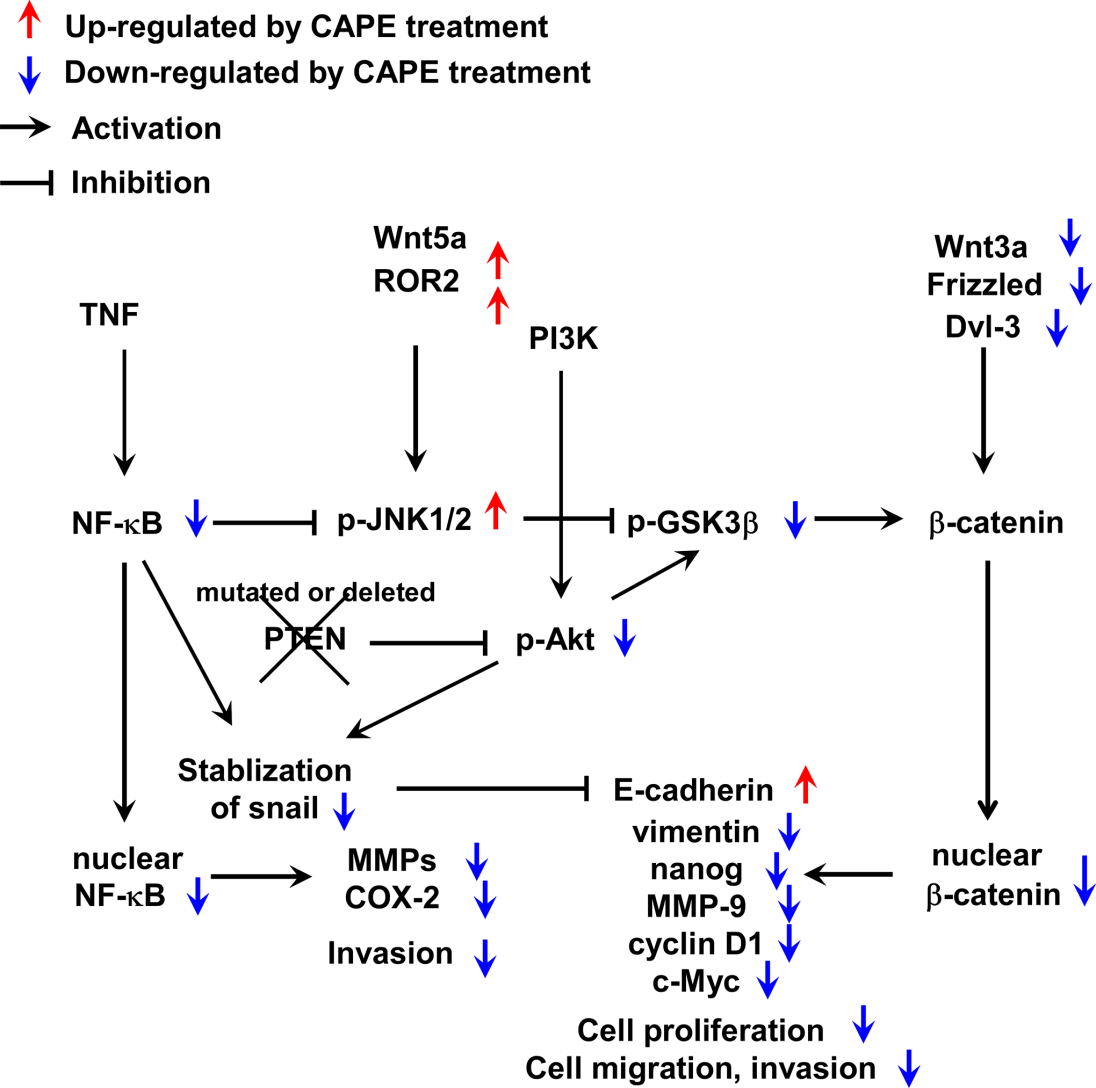
A summary of signaling pathways affected by CAPE treatment in PC-3 and DU-145 cells Red arrows indicate signaling proteins being elevated by CAPE treatment, while blue arrows indicate signaling being suppressed by CAPE treatment.

We have generated a summary of signaling pathways affected by CAPE treatment (Figure 9). CAPE treatment reduces abundance and activity of NF-κB while elevates abundance of ROR2 ad Wnt5a. This in turn increased phosphorylation of JNK1/2, which decreased the phosphorylation of GSK-3β. CAPE also suppressed the protein level of Wnt3a, Frizzled proteins, and Dvl-3, resulting in the reduction of nuclear β-catenin. CAPE lessens the phosphorylation of Akt, thus reduces the stability of Snail. CAPE also increases E-Cadherin and decreases vimentin, nanog, MMP-9, cyclin D1, c-Myc, and COX-2. These changes therefore suppress the cell proliferation, migration, and invasion of PCa cells.

In conclusion, CAPE treatment suppresses PCa metastasis via activation of non-canonical Wnt signaling as well as inhibition of canonical Wnt signaling, EMT, and NF-κB signaling pathway. This is the first report revealing that CAPE treatment can stimulate non-canonical Wnt signaling but suppress canonical Wnt signaling pathways. We believe that CAPE may be a potential therapeutic agent for advanced PCa.

## MATERIALS AND METHODS

### Chemicals

All chemicals used in this research were purchased from Sigma-Aldrich (St. Louis, MO, U.S.A.).

### Cell Culture

PC-3 and DU-145 cells were purchased from Bioresource Collection and Research Center (Hsinchu city, Taiwan). DU-145 and PC-3 cells were maintained in Dulbecco's Modified Eagle's Media (DMEM) contain 10% FBS, penicillin (100 U/ml), and streptomycin (100 μg/ml) at 37°C with 5% CO_2_ and replace fresh medium once every two days.

### Plasmids

Expression of hairpin shROR2 plasmid (pGPU6-RFP-shROR, target sequence: CCAGCCAA GACATGGAAAT) and its control vector (pGPU6-RFP-shNC, target sequence: TTCTCCGAACGTGTCACGT) were purchased from GeneDireX (Hsinchu City, Taiwan).

### Transwell migration assay

Migration assays for PC-3 and DU-145 cells were performed following the instructions of the transwell kit purchased from BD Bioscience (Franklin Lakes, NJ, U.S.A.) and as previously described [44]. PC-3 and DU-145 cells seeded at a concentration of 2 × 10^4^/250 μl into upper compartment of transwell. The lower compartment of transwell was filled with 800 μl complete medium. PC-3 and DU-145 PCa cells were pre-treated with 0, 20, 40 and 80 μM CAPE for 24 h. After appropriate incubation time, total number of migrated cells was counted following standard procedures. Cells were then removed from tissue culture plates with trypsin, and washed once with DMEM serum free medium.

### Transwell invasion assay

An invasion assay with PC-3 and DU-145 PCa cells was performed with Growth Factor Reduced BD BioCoat Matrigel invasion chambers according to the manufacturer's instructions and as previously described [44]. PC-3 and DU-145 cells seeded at a concentration of 2 × 10^4^/500 μl into into upper compartment of transwell. The lower compartment of transwell was filled with 800 μl complete medium. PC-3 and DU-145 PCa cells were pre-treated with 0, 20, 40 and 80 μM concentrations of CAPE for 24 h. After appropriate incubation time, total number of migrated cells was counted following standard procedures.

### Wound healing assay

Wound healing assay with PC-3 and DU-145 PCa cells was performed with ibidi culture insert (Applied Biophysics, Troy, NY, U.S.A.) according to the manufacturer's instructions and as previously described [44]. Briefly, PC-3 or DU-145 cells that pre-treated with different concentration of CAPE for 24 h seeded at a concentration of 3.5 × 10^4^/100 μl into individual compartment of ibidi culture insert overnight. We filled the culture plate with DMEM complete medium and then removed the ibidi culture inserts. Cell migration was monitored once per two hours by photographing with a live imaging microscope (Leica AF 6000 LX, Leica, Wetzlar, Germany). The results of wound closure was captured and displayed as photographs.

### Gelatin zymography assay

Gelatin coated poly-acrylamide gel electrophoresis for MMP-2 and MMP-9 activity assay was performed as previously described [45]. DU-145 and PC-3 cells were pre-treated with different concentration of CAPE (0, 20, 40 and 80 μM) for 24 h. Briefly, one million of DU-145 or PC-3 cells seeded onto 10 cm dish overnight, and then treated cells with different concentration of CAPE (0, 20, 40 and 80 μM) for 24 h. Culture medium contained CAPE was exchanged with fresh 5 ml serum-free DMEM and incubated with additional 18 h for MMPs secretion. Each conditional medium was collected and concentrated by Vivaspin concentrator (GE Healthcare). The activity of MMP-2 and MMP-9 were determined through (1 mg/ml) gelatin coated PAGE, refolding with 2.5% triton X-100 and incubated in development buffer (25 mM Tris-HCl pH7.5, 150 mM NaCl and 5 mM CaCl_2_) at 37°C for 48 h. Image of each signal intensity after stained gel by Commassie Brilliant Blue R-250 procedures.

### Micro-Western Arrays (MWA)

PC-3 and DU-145 PCa cells were treated with vehicle (ethanol) or different concentration of CAPE (20, 40 and 80 μM) in DMEM with contain 10% FBS for 24 h. The MWA were conducted to measure protein expression and modification as previously described [9]. Detection of α-tubulin and β-actin were used as loading control. Scanned images were obtained by using Odyssey Infrared Imaging System. Intensity of bands for different proteins was quantified with Odyssey 3.0 software. All the antibodies used in present study were listed in Supplementary Material file.

### Western blotting analysis

All the antibodies used in present study were listed in Supplementary Material file. Anti-rabbit and anti-mouse IgG secondary antibodies purchased from Santa Cruz (Santa Cruz, CA, U.S.A.). Expression of α-tubulin and β-actin were used as loading control. Intensity of bands for different proteins was quantified with Image J 1.48 software after EPSON stylus TX130 scanning.

### TCF-LEF reporter assays

The TCF/LEF Reporter kit is designed to monitor the activity of Wnt signal transduction pathways in cultured cells. For measuring TCF-LEF activity, 2×10^5^ cells were seeded per well in 12-well plate for 24 h. PCa cells were then co-transfected with 1 ng of phRL-CMV-Renilla luciferase plasmid and 1 μg of pGL4.49[luc2P/TCF-LEF RE/Hygro] Vector using the PolyJet™ DNA *In Vitro* transfection reagent. The ratio about PolyJet^™^ and plasmid was 3μl transfection reagent to 1 μg DNA. Six hours after transfection, cells were treated with 0, 20, 40 and 80 μM of CAPE for 24 h and were lysed in 100 μl passive lysis buffer (Promega, Madison, WI, U.S.A.). Luciferase activity was measured using a Dual-Luciferase kit (Promega) in a Monolight luminometer (BD Biosciences).

### Xenografts in athymic mice

For the hind leg prostate cancer metastasis model, 6–8 weeks old male Balb/c nu/nu mice (purchased from BioLASCO, Taipei, Taiwan) were injected with 2.5 × 10^5^ PC-3^luc^ cells stably expressed firefly's luciferase into right side hind leg muscle for cancer invasion study. For the tail vein injection model, 1 × 10^6^ PC-3^luc^ cells were injected into mice tail vein. CAPE (15 mg/kg in 0.1 ml 50% DMSO) or vehicle (0.1 ml 50% DMSO) was administered every three days to mice by i.p. starting one week after the tumor cell inoculation. Image of the xenografts in the nude mice was monitored weekly using *in vivo* bioluminescence IVIS imaging system Xenogen IVIS-200 (Caliper Life Sciences, Hopkinton, MA, U.S.A.).

### Immunohistochemistry

Paraffin embedded tissue sections derived from tumor bearing mice were deparaffinized with xylene, rehydration with graded concentrations of ethanol, and then practice antigen recovery with 10 mM citrate buffer (pH 6.0) via Heat-Induced Epitope Retrieval (HIER) method, following by blocking endogenous peroxidase with 3% H_2_O_2_ in TBS for 15 min. Samples were blocked with Ultra V block (Thermo Scientific, Waltham, MA, U.S.A.) and incubated with specific antibodies at a 1:100 dilution for overnight at 4°C. The tissue sections were rinsed with TBST three times per five minutes and then incubated with HRP conjugated antibodies (Santa Cruz Biotechnology, Inc.). Excess antibodies were removed by rinsing with TBST three times per five minutes and tissue specimens were then reacted with DAB chromogen and substrate mixture (Thermo Scientific) for appropriate timing. Immunostaining was visualized after couterstaining with hematoxylin. All of antibodies used in present study were listed in Supplementary Material file.

### Public domain data

Data were downloaded from Oncomine (http://www.oncomine.com) and PubMed GEO profile. Expression profiles of *NFKBIA* gene expression analysis was extracted from Holzbeierlein prostate (Affymetrix U95 human gene) [46], which contains 40 primary prostate tumors and 9 metastatic prostate tumors from patients received radical prostatectomy. Expression profile of *RELA* gene was extracted from Grasso prostate datasets (Agilent Human Genome 44K) [47], which contains 51 primary prostate tumors and 32 metastatic prostate tumors. Expression profiles of *SNAI1* and *DUSP6* genes expression analysis were extracted from Chandran prostate dataset (GSE6752) (Affymetrix GeneChip HGU95av2, HGU95b and HGU95c arrays) [48], which contains 10 primary prostate tumors and 21 metastatic prostate tumors. Expression level of *ROR2* (Reporter: GPL8300, 212_at), *FZD5* (Reporter: GPL8300, 34997_r_at) and *SMAD4* (Reporter: GPL8300, 510_g_at) were extracted from GDS2545 on PubMed GEO profile which contains 18 normal prostate epithelial tissue, 65 primary prostate tumors and 25 metastatic prostate tumors. Expression level of *DVL3* was extracted from GDS1439 on PubMed GEO profile (reporter: GPL570, 201908_at) which contains 6 benign prostate tissue, 7 primary prostate tumors and 6 metastatic prostate tumors; and *PLCB3* gene expression was extracted from GDS2547 (reporter: GPL93, 63936_at) which contains 64 primary prostate tumors and 25 metastatic prostate tumors.

### Data analysis

Data were presented as mean ± SD of at least three independent experiments or are representative of experiments repeated more than three times. Student's *t* test (two-tailed, paired) was used to evaluate the statistical significance of results from transwell migration and invasion assays.

## SUPPLEMENTARY MATERIALS FIGURE


