# Supplementary material for: CAPE suppresses migration and invasion of prostate cancer cells via activation of non-canonical Wnt signaling
Source: Oncotarget. 2016 May 15;7(25):38010–24. doi: 10.18632/oncotarget.9380 (PMC5122368; doi:10.18632/oncotarget.9380)
Supplement: Supplementary file 1 [file oncotarget-07-38010-s001.pdf]

CAPE suppresses migration and invasion of prostate cancer cells via activation of non-canonical Wnt signaling

Supplementary Materials

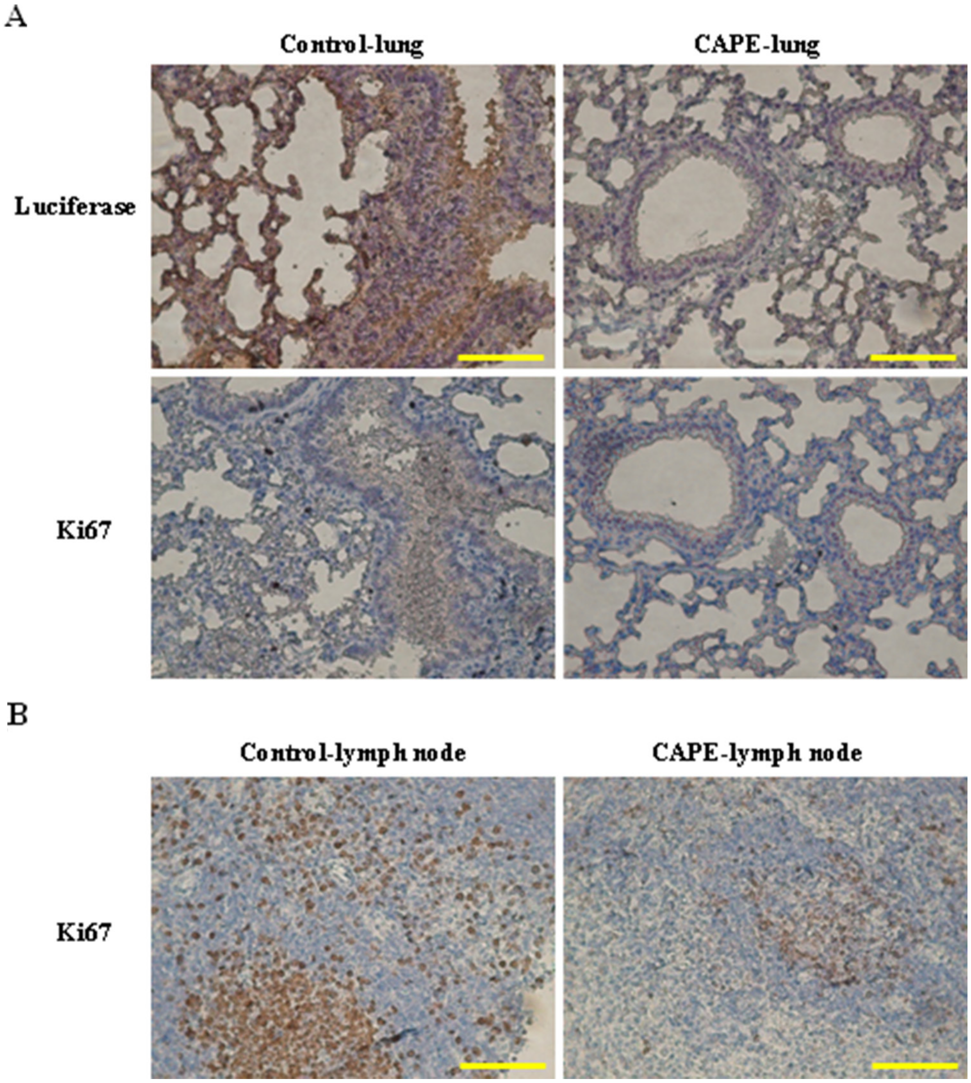

Supplementary Figure S1: CAPE treatment suppressed cancer metastasis of PC-3 xenografts toward lung and lymph node in nude mice. Immunohistochemistry staining was performed to determine levels of Ki67 in PC-3 tumors metastasized to (**A**) lung and (**B**) lymph node of nude mice from Figure 5. Scale bars represent 100 µm at 400× magnification.

SUPPLEMENTARY MATERIAL

Antibodies used in Micro-Western Array experiment

Antibodies of E-Cadherin, and N-Cadherin were from BD Biosciences (Franklin Lakes, NJ, U.S.A.). Antibodies of ROR2, and Snail were from Biorbyt (Berkeley, CA, U.S.A.) Antibodies of Akt, phospho-Akt Ser473, phospho-Akt Thr308, caspase-3, b-catenin, cdc2, phospho-cdc2 Tyr15, phospho-CREB Ser133, CrkL, phospho-CrkL Tyr207, Cyclin B1, Cyclin D1, cyclin E, phospho-cyclin E Thr62, EGFR, FAK, Gab1, GSK3α, phospho-GSK3α Ser21, GSK3β, phospho-GSK3β Ser9, Hamartin/TSC1, Jak1, phospho-Jak1 Tyr1022/1023, Jak2, phospho-Jak2 Tyr1007/1008, phospho-p44/42 MAPK (Erk1/2) Thr202/Tyr204, phopsho-p38 MAPK Thr180/Tyr182, mTOR, phospho-mTOR Ser2481, c-Myc, Nanog, NF-κB p65, NF-κB1 p105/p50, p21Cip1, $p27^{Kip1}$, p70 S6 Kinase, phopsho-PDGFRα Tyr849/PDGFR β Tyr857, phospho-PDGF Receptor α Tyr1018, phospho-PDGFR β Tyr751, phospho-PDGFR β Tyr771, phospho-PDGFR β Tyr1009, phospho-PDGFR β Tyr1021, PDK1, phospho-PDK1 Ser241, c-Raf, Rb, phospho-Rb Ser780, phospho-Rb Ser807/811, phospho-Ros Tyr2274, SAPK/JNK, phospho-SAPK/JNK Thr183/Tyr185, Shc, phospho-Shc Tyr317, SHP-1, SHP-2, Snail, Src, phospho-Src Tyr527, Syk, Tuberin/TSC2, phospho-Tuberin/TSC2 Thr1462, phospho-VEGFR 2 Tyr1059, phospho-VEGFR 2 Tyr1175, phospho-VEGFR 2 Tyr1212, and phospho-VEGFR 2 Tyr951 were from Cell Signaling (Danvers, MA, U.S.A.). Antibodies of Acetyl CoA Carboxylase 1, E-Cadherin, N-cadherin, pro-caspase-3, caspase-8, caspase-9, β-catenin, Claudin 1, Claudin 2, CREB1, DKK1, FAK, phospho-FAK Tyr397, phospho-FAK Tyr576/Tyr577, Fibronectin, GAPDH. HNF4α, IκBα, phospho-IκBα Ser32, phospho-IκBα Ser36, phospho-IκBα Tyr42, IKKα, IKKβ, IKKe, phospho-MEK1 Ser298, MEK2, MKP-3, c-Myc, phospho-NF-κB p65 Ser529, phospho-NF-κB p65 Ser536, NF-κB p100, PIAS1, PIAS2, PIAS3, PLCβ III, RELB, RHEB, RIPK1, ROR2, phospho-SHP-2 Tyr542, phospho-SHP-2 Tyr582, SLUG, Smad1, Smad2, Smad3, phospho-Smad3 Ser423/Ser425, Smad4, Smad5, phospho-Smad5 Ser463/Ser465, phospho-Smad5 Ser463/Ser465, Smad7, Smad9, Smurf2, TAB1, TAB3, TRAF2, TRAF3, TRAF4, TRAF6, and vimentin were from Epitomics (Burlingame, CA, U.S.A.). Antibodies of Akt3, Cdc25A, and Cdc4 were from Genetex (Hsinchu, Taiwan). Antibodies of Acetyl CoA Carboxylase 1, Akt1, Akt2, Akt3, Akt3, Bad, Bak, Bcl-2, Bcl-3, Bcl-6, Bid, Bin1, phospho-BMK1/Erk5 Thr218/Tyr220, phospho-Cdc25C Ser216, caspase-2, Cathepsin K, Caveolin, Cdk4, Cyclin A, cyclin B1, cyclin E1, cyclin E2, E2F-1, EGFR, phospho-EGFR Tyr845, phospho-EGFR Tyr1045, phopsho-EGFR Tyr1069, phospho-EGFR Tyr1086, phospho-EGFR Tyr1148,phospho-EGFR Tyr1173, eNOS/NOS III, phospho-eNOS/NOS III Ser116, ErbB-2/HER-2, phospho-erbB2 Thr686, phospho -erbB2/Her2 Tyr1112, phospho -erbB-2/HER-2 Tyr1248, phospho -FAK Ser722, phospho-FAK Tyr861, Frizzled 5, phospho-Glycogen Synthase Ser641/Ser645, phospho-GSK3 Tyr279/Tyr216, HIAP-2, HIF-1α, IL-1R1, Insulin Receptor beta subunit, phospho-Insulin Receptor Tyr1322, MAPK1/2, phospho-MAP Kinase1/2 (Erk1/2), Maspin, Mcl-1, phospho-MEK1 Ser218/222/MEK2 Ser222/226, phospho-MEK1 Ser298, phospho-MEK1 Thr292, phospho-MEK1 Thr386, phospho-mTOR Thr2446, phospho-c-Myc Thr58/Ser62, MyD88, NF-κB p50, NF-κB p50, NF-κB p65, nNOS/NOS I, phospho-nNOS/NOS I Ser1417, phospho-p70 S6 Kinase Thr389, phospho-p70 S6 Kinase Thr389, phospho-p70 S6 Kinase Thr412, phosphor-p70 S6 Kinase Thr421/Ser424, PI3K p85, PI3K p110α, PI3K p110β, PI3K p110γ, PI3K p110δ, phospho-PDGFRα Tyr754, phospho-PDGFRβ Tyr716, PPARα, PPARγ, P-Selectin Glycoprotein Ligand-1, phospho-Raf1 Ser43, phospho-Raf1 Ser259, phospho-Raf-1 Tyr340/Tyr341, phospho-Raf-1 Ser621, c-Rel (NFκB), RIAP-3, S-100 Protein, phospho-SHC Tyr239, phospho-SHP-2 Ser576, Smac/DIABLO, phospho-Src Tyr416, phospho-Src Tyr418, SREBP1, SREBP2, phospho-STAT1 Ser727, phospho-STAT2 Tyr689, phospho-STAT5A/B Tyr694/699, phospho-STAT6 Tyr641, Talin, TLR6, TNF Receptor, tPA, and phospho-TRF2 Thr188 were from Millipore (Billerica, MA, U.S.A.). Antibodies of b-actin, Akt1, Akt2, claudin 3, claudin 4, and GAPDH were from Novus (Littleton, CO, U.S.A.). Antibodies of BMP4, B-RAF, CD36, b-catenin, GRB2, IL3, IL-6, OSM, SOCS3, and a-tubulin were from Origene (Rockville, MD, U.S.A.). Antibodies of Frizzled 4 was from R & D (Minneapolis, MN, U.S.A.). Antibodies of phospho-cyclin E Thr395, MEK-1, phospho-MEK-1 Ser222, phospho-MEK-1 Thr291, phospho-MEK-1 Thr386, phospho-MEK-2 Thr394, MEK-3

phospho-MEK-3 Ser189, MEK-4, phospho-MEK-4 Ser80, phospho-MEK-4 Thr261, MEK-5, MEK-6, MEK-7, phospho-MEK-6 Ser207, MEK kinase-1, phospho-MEK kinase-1 Thr1402, MEK kinase-2, MEK kinase-3, phospho-MEK kinase-3 Ser166, MEK kinase-4, NFκB p65, and p21Cip were from Santa Cruz (Santa Cruz, CA, U.S.A.). Antibodies of Ki67, NF-κB p65, and vimentin were from Thermo (Waltham, Massachusetts, U.S.A.)e.
